# Acquired Thrombotic Thrombocytopenic Purpura (TTP) Presenting With Synthetic Cannabinoid Use

**DOI:** 10.7759/cureus.61536

**Published:** 2024-06-02

**Authors:** Megan B Douglass, Olivia Yessin, Joseph Elengickal, Sheldon Charpenter, Cayla Campbell

**Affiliations:** 1 Internal Medicine, Prisma Health Richland Hospital, Columbia, USA

**Keywords:** altered mental state, : acute kidney injury, microangiopathic hemolytic anemia (maha), synthetic cannabinoid, thrombotic thrombocytopenic purpura

## Abstract

Synthetic cannabinoids (SCs) have become commercially available throughout the United States as manufacturers circumvent regulations with labels stating “not for human consumption” with misleading advertisements, resulting in the consumption of products that are not safe or regulated. We present a case report of a middle-aged woman exhibiting altered mental status secondary to SC use who was found to have severe thrombocytopenia and hemolytic anemia. She was later confirmed to have thrombotic thrombocytopenic purpura (TTP) through ADAMTS13 testing. TTP is one of several platelet-related disorders presenting with findings of hemolytic anemia and thrombocytopenia. The presence of altered mental status is typically used as a symptomatic differentiator between hemolytic uremic syndrome, immune thrombocytopenic purpura, and TTP. SCs can cause superimposed altered mental status, which, in the setting of a concomitant platelet disorder, can complicate the standard workup and prolong the time to a final diagnosis. This case serves as an essential reminder that collecting detailed social history and promptly recognizing laboratory abnormalities is critical for early recognition of TTP, as the diagnosis is time-sensitive and delays in recognition can lead to significant morbidity and mortality.

## Introduction

Synthetic cannabinoids (SCs), also known as “K2” or “Spice,” are classified by the United States Drug Enforcement Administration (DEA) as class I controlled substances [1], with many specific SCs considered illegal. However, manufacturers of SCs circumvent these regulations by creating new products with different ingredients or by using the label “not for human consumption,” allowing them to be sold in stores despite the legal restrictions [2]. 

SCs are a group of substances often used as recreational drugs due to their psychoactive effects. They are chemically diverse and have more significant toxicity and potential for addiction than natural cannabinoids due to SCs binding more securely to the cannabinoid-1 (CB-1) receptors. Several of the physiologic effects of SCs are similar to marijuana, including conjunctival injection, tachycardia, and ataxia. Despite these comparisons, marijuana contains natural cannabinoids, while SCs are chemically synthesized compounds that are not structurally similar to marijuana and can result in life-threatening chemical manifestations. Anecdotal reports have identified very severe side effects of SCs, including psychosis, cardiac arrhythmias, electrolyte abnormalities, convulsions, and panic attacks [3]. A report from a multicenter registry of medical toxicologist consultations found that of the 277 consulted patients reporting the use of SCs, 4% of those patients had an acute kidney injury (AKI) [4]. SCs were found to cause neurologic and cardiovascular side effects more commonly [4]. Although rare, a handful of case reports have documented an AKI and thrombotic microangiopathy (TMA) secondary to SC usage [5]. 

Thrombotic thrombocytopenic purpura (TTP) is a thrombotic microangiopathy characterized by reduced activity of ADAMTS13, a protease responsible for cleaving von Willebrand factor (VWF). VWF plays a vital role in facilitating the adhesion of platelets to exposed collagen and platelet aggregation. When ADAMTS13 activity is low, uncleaved multimers of VWF can form clots in small blood vessels, ultimately leading to end-organ damage [6]. While TTP is classically characterized by a symptomatic pentad of fever, thrombocytopenia, microangiopathic hemolytic anemia (MAHA), renal dysfunction, and neurologic dysfunction, it rarely presents with all five symptoms. Additional presenting symptoms can include dyspnea, weakness, fatigue, bruising, nausea, and vomiting [6].

TTP can be hereditary or immune-mediated. Hereditary TTP, resulting from mutations in ADAMTS13, is uncommon and does not necessarily result in a clinically significant deficiency until an inciting event occurs. Immune-mediated TTP develops from the formation of autoantibodies against ADAMTS13. Immune-mediated TTP can be further classified as drug-induced [6]. Based on the Oklahoma TTP-hemolytic uremic syndrome (HUS) Registry, the incidence of immune-mediated TTP is three per one million adults per year, and the median age of presentation is 40 years [7]. TTP is more likely to present in women, African Americans, and patients with a body mass index greater than 40 [6]. Without prompt treatment, TTP is often fatal. Immediate plasmapheresis should be initiated to reduce mortality when there is a high degree of clinical suspicion for TTP after a hematology-oncology consultation [8].

## Case presentation

A 56-year-old woman with a past medical history of seizures, asthma, tobacco use, and recreational marijuana use was brought to the emergency department by her partner for several days of altered mental status after using a vape containing synthetic marijuana. Due to the patient's agitation and altered mentation, her partner provided her medical history. He stated that three to four days before her presentation, the patient had used for the first time a vape pen containing synthetic marijuana purchased from a local store. Shortly after this, she began to exhibit unusual behavior, including disorientation, speech impairment with marked word-finding difficulty as well as severe agitation. This prompted her partner to seek medical attention at a local urgent care, where the patient received intravenous (IV) fluids but refused most laboratory studies. The patient's partner reported that her mentation transiently improved; however, she became agitated and disoriented again the following morning. In addition, over this period, she had several episodes of grossly bloody urine. 

Upon arrival at our emergency department, the patient was afebrile (97.8 ℉), tachycardic (120 beats per minute), and hypertensive (143/107). On our assessment, she was disoriented, unable to respond to questions, and required mechanical restraint due to her excessive agitation. No focal neurologic deficits were immediately noted. Scattered bruising and excessive drooling were noted. A computerized tomography (CT) scan of the head revealed no acute intracranial abnormalities. 

Admission labs were significant for anemia and thrombocytopenia with hemoglobin 9.1 g/dL and platelet count 36 x 10^9^/L, notably reduced from her platelet count six months earlier of 330 x 10^9^/L. She was seen to have a marked reticulocytosis (7.99%). She was also noted to have elevated direct and total bilirubin at 2.2 mg/dL and 3.1 mg/dL, respectively. Lactate dehydrogenase (LD) was elevated at 1,189 U/L, and haptoglobin level was significantly decreased (<8 mg/dL), suggestive of intravascular hemolysis. Renal function was notably normal on admission. The patient's prothrombin time (PT) and international normalized ratio (INR) were mildly elevated at 18.9 and 1.5, respectively (Table 1). Visual analysis of the patient's blood smear revealed numerous schistocytes, confirming the presence of hemolytic anemia. The patient's PLASMIC score was elevated at 7, corresponding to a 72% Risk of severe ADAMTS13 deficiency.

**Table 1:** Laboratory test results with reference values D0 indicates the value at the time of presentation. D3 represents the value on hospital day three. D14 indicates the value on hospital day 14, when the patient was discharged.

Pertinent Lab Results	Case Presentation Values	Normal Range
Hemoglobin	9.1 g/dL	Male: 14-18 g/dL Female: 12-16 g/dL
Platelet Count	D0: 36 x 10^9^/L	150-400 × 10^9^/L
	D3: 160 x 10^9^/L	
	D14: 220 x 10^9^/L	
Reticulocyte	7.99%	0.5-2.5%
Direct Bilirubin	2.2 mg/dL	0.1-0.3 mg/dL
Total Bilirubin	3.1 mg/dL	1.0-12.0 mg/dL
Lactate Dehydrogenase	1,189 U/L	140-280 U/L
Haptoglobin	<8 mg/dL	41-165 mg/dL
Prothrombin Time (PT)	18.9 seconds	11-13.5 seconds
International Normalized Ratio (INR)	1.5	1.1 or below
ADAMTS13 Activity	<5%	50-160%

Given the patient's presentation with hemolytic anemia and thrombocytopenia in conjunction with acute encephalopathy, a provisional diagnosis of TTP was made, and treatment was promptly initiated. She was urgently transfused fresh frozen plasma (FFP) as a temporizing measure. Subsequently, she was admitted to the intensive care unit (ICU), where she was intubated for emergent line placement, given her severe agitation. She underwent plasmapheresis (PLEX) immediately following line placement and was additionally initiated on prednisone 60 mg. A second round of PLEX was performed the following morning (hospital day two). By then, the patient was alert and able to follow commands and nod her head, though she remained intubated. She was extubated later that day as her encephalopathy had resolved. The patient received three days of PLEX, with the recovery of her platelet count to 160 x 10^9^/L by the third day. She was transferred out of the ICU and discharged on hospital day six on a 14-day course of oral prednisone. Her platelet count had recovered to 220 x 10^9^/L at discharge. The patient's ADAMTS13 activity drawn on the day of admission ultimately resulted as <5%, and screening for ADAMTS13 inhibitor was positive, confirming a diagnosis of acquired TTP. In our patient's case, the use of SCs was thought to be the precipitant of her acquired TTP. 

## Discussion

While drug-induced TTP is a rare but relatively recognized entity, there are few existing case reports to our knowledge of TTP or other thrombotic microangiopathies precipitated by SC use. Several case reports of SC-induced idiopathic thrombocytopenic purpura (ITP) have been reported in the literature, including a 2014 case report by Ozturk et al. [9]. TTP-like illness has also been reported in several cases of IV drug use [1] although our patient was not using IV drugs to our knowledge. The first case of biopsy-proven thrombotic microangiopathy with concomitant SC usage was reported by Karass et al. in 2017 and was thought to be similar in pathophysiology to a hemolytic uremic syndrome. 

The mainstay of TTP treatment includes therapeutic plasma exchange (TPE), glucocorticoids, rituximab, and caplacizumab. TPE removes auto-antibodies for ADAMTS13 and provides ADAMTS13 from donor plasma. Restoration of ADAMTS13 allows VWF multimers to be properly cleaved, preventing microvascular thrombosis and organ damage. Monoclonal antibodies can be incorporated into treatment after TPE [8].

Treatment of TTP is highly time-sensitive, and mortality strongly depends on time to therapeutic intervention. In cases like ours, where access to plasmapheresis will present a delay, several temporizing measures, including transfusion of FFP or use of the monoclonal antibody caplacizumab, can be performed until TPE can be initiated [8].

## Conclusions

This case of acquired TTP, likely precipitated by SC usage, represents an important differential diagnosis that should be included for patients presenting with altered mental status in the context of recent synthetic marijuana usage. This diagnosis should be made through a careful history and physical exam, as well as analysis of labs and peripheral blood smear. Severe acute thrombocytopenia and hemolytic anemia in the setting of SC usage should prompt immediate concern for thrombotic microangiopathy, including drug-induced TTP, and warrant emergency hematology oncology consultation. While ADAMTS13 testing is taking place, TPE should not be delayed if the provider has high clinical suspicion for TTP. If TPE is not immediately available, FFP and caplacizumab can temporarily benefit the patient until TPE can be initiated. 
